# Emotional Disorders in Pairs of Patients and Their Family Members during and after ICU Stay

**DOI:** 10.1371/journal.pone.0115332

**Published:** 2015-01-23

**Authors:** Renata Rego Lins Fumis, Otavio T. Ranzani, Paulo Sérgio Martins, Guilherme Schettino

**Affiliations:** 1 Intensive Care Unit, Hospital Sírio Libanês, Rua Dona Adma Jafet, 91, São Paulo 01308-050, Brazil; 2 Respiratory Intensive Care Unit, Pulmonary Division, Heart Institute, Hospital das Clínicas, University of São Paulo, Rua Dr. Enéas de Carvalho Aguiar, 255, São Paulo 05403-900, Brazil; Central Institute of Mental Health, GERMANY

## Abstract

**Introduction:**

Patients and family members undergo different experiences of suffering from emotional disorders during ICU stay and after ICU discharge. The purpose of this study was to compare the incidence of anxiety, depression and post-traumatic stress disorder (PTSD) symptoms in pairs (patient and respective family member), during stay at an open visit ICU and at 30 and 90-days post-ICU discharge. We hypothesized that there was a positive correlation with the severity of symptoms among pairs and different patterns of suffering over time.

**Methods:**

A prospective study was conducted in a 22-bed adult general ICU including patients with >48 hours stay. The Hospital Anxiety and Depression Scale (HADS) was completed by the pairs (patients/respective family member). Interviews were made by phone at 30 and 90-days post-ICU discharge using the Impact of Event Scale (IES) and the HADS. Multivariate models were constructed to predict IES score at 30 days for patients and family members.

**Results:**

Four hundred and seventy one family members and 289 patients were interviewed in the ICU forming 184 pairs for analysis. Regarding HADS score, patients presented less symptoms than family members of patients who survived and who deceased at 30 and 90-days (p<0.001). However, family members of patients who deceased scored higher anxiety and depression symptoms (p = 0.048) at 90-days when compared with family members of patients who survived. Patients and family members at 30-days had a similar IES score, but it was higher in family members at 90-days (p = 0.019). For both family members and patients, age and symptoms of anxiety and depression during ICU were the major determinants for PTSD at 30-days.

**Conclusions:**

Anxiety, depression and PTSD symptoms were higher in family members than in the patients. Furthermore, these symptoms in family members persisted at 3 months, while they decreased in patients.

## Introduction

Survivors of Intensive Care Units (ICUs) may experience psychological distress for a long time after ICU discharge [1–3]. Usually, patients and family members suffer from symptoms of anxiety, depression and post-traumatic stress [1–7].

Over the last decade, family-centered care, in which close attention is paid to communication and the emotional needs of family members, has been studied aiming to reduce stress, with special attention to families of dying patients [8–10]. Recently, many ICUs are changing their restrictive visitation policy to an open visitation one, in order to meet the family needs to stay together with the patient, reduce their distress and improve family satisfaction [11–17].

Literature shows that family members of critically ill patients, particularly spouses, suffer relevant psychological distress and that they need support during the follow-up period [5,6,18]. The importance of this care, including follow-up, is a recognized component of good quality care [7,8,19].

Earlier publications have identified emotional disorders in patients and family members during and after discharge from intensive care and that increased rates of anxiety and depression during ICU stay was associated with a severe post-traumatic stress reaction [1,2,7,20]. Others factors, such as the patient severity assessed by the SAPS 3 score, patient death, patient age and lack of information, were predictors of psychological distress in family members [7,8]. Among patients, their experiences during ICU stay and demographics variables were predictors of PTSD [2]. The complexity of patients with illness severity requiring invasive therapy such as mechanical ventilation, renal replacement therapy, extracorporeal membrane oxygenation, prolonged ICU stay may represent a PTSD risk factor. Also, family members of seriously ill patients, facing risk of poor prognosis, or whose relative died in the ICU were associated with anxiety, depression and PTSD [4,7,21].

Psychological distresses have been discussed with considerable attention in recent years, but to date, few studies have addressed the symptoms of anxiety, depression and post-traumatic distress in the pair, patient and family member, including ICU stay and follow-up [18,22,23]. Literature suggests that patients and their family members may experience different levels of anxiety, depression and post-traumatic distress in varied ICU recovery periods [18,22,23]. We hypothesized that there was a positive correlation between the patient’s severity of symptoms and suffering of the family member and that should be taken into account when analyzing each pair. The purpose of this study was to compare the incidence of anxiety, depression and post-traumatic stress symptoms in pairs (patient/respective family member) during ICU stay and at 30 and 90-days post ICU discharge.

## Materials and Methods

### Setting

This prospective study was conducted in a tertiary private teaching hospital, Hospital Sírio-Libanês, in São Paulo, Brazil. The institutional review board (IRB), called the Comitê de Ética em Pesquisa da Sociedade Beneficente de Senhoras do Hospital Sírio Libanês, reviewed and approved this study (HSL – protocol number 2010/44). All patients and their respectively family members with a more than 48 hours ICU stay were invited to participate and sign a written informed consent. The study was conducted in a medical-surgical unit, comprising 22 private rooms. The professional-to-bed ratios in the ICU are: nurse 1:4; nurse-assistant 1:2; physician 1:6 (day shift) and 1:10 (night shift).

The ICU has a 24-hour visitation policy with family facilities to encourage the visit (day or night free entry, with possibility to change the visitor at any time, option to sleep with the patient in an individual box with sofa, TV and bathroom).

### Subjects of study

Inclusion criteria for patients were age over 18 years and more than 48 hours of ICU stay. Exclusion criteria for patients were: psychiatric disorders, severe neurologic disease, too ill to answer at the first assessment or with any difficulty for follow-up due to their physical impairment or limitations (e.g. hearing, incapacity to speak, language barriers, too old to participate). For family members, we included only one per patient, the closest next-of-kin (spouse, child, parents, sisters), identified as the family member most likely to be involved with the patient’s care. Exclusion criteria for family members were psychiatric disorders. For patients and family members, psychiatric disorders included anxiety and depression under drug treatment at ICU admission.

After 48 hour of ICU stay, both patient and their family member were approached. If the patient was unable to participate at first assessment, because of clinical conditions (e.g. mechanical ventilation, delirium), only the family member was interviewed. During ICU stay, when patients were able to participate, they were assessed and interviewed. At 30 and 90-days, only those who participated in the ICU period were followed up.

### Interviews

Patients and their family members were interviewed while in the ICU using the Hospital Anxiety and Depression Scale (HADS). At 30 and 90 days after ICU discharge they were also interviewed by phone to complete the Impact of Event Scale (IES) and HADS.

HADS score for each subscale (anxiety and depression) ranges from 0–21 and a cut-off of > 10 was used to depict each condition [4–6]. Scores for the entire scale (emotional distress) range from 0–42, with higher scores indicating more distress.

The IES score has been used for many years and seems reliable across a broad range of traumatic events and it can be easily carried out during a telephone interview. The IES is not a tool for diagnosing PTSD, however it detects symptoms indicating risk of PTSD. It comprises 15 items, seven of which measure intrusive symptoms (for example, intrusive thoughts, nightmares, intrusive feelings and imagery) and eight measure avoidance symptoms (numbing of responsiveness, avoidance of feelings, situations, and ideas). Respondents are asked to rate the items according to how often each has occurred in the past 7 days. The IES score ranges from 0–75 points with higher scores indicating more severe post-traumatic stress symptoms. Patients were classified as having low or high IES scores using 30 as the cutoff in agreement with previous reports that higher than 30 points indicates post-traumatic stress reaction with a significant risk of PTSD [7,8]. To ensure optimal quality of the data, all interviews were conducted by the same person (RRLF), a psychologist with ICU interview experience [24]. Both scales were previously validated in Brazil [25,26].

### Data collection

For each patient the following information was collected: age, gender, marital status, level of education, cause of ICU admission, cancer disease, SAPS 3, Glasgow, SOFA, ICU length of stay (LOS), need of mechanical ventilation, renal replacement therapy in the ICU, delirium (positive CAM-ICU) and final outcome in the ICU. The following information was supplied by the family member: gender, age, marital status, level of education, religion belief, relationship with the patient, previous ICU experience and how much time per day the family member spends visiting patient in the ICU.

### Statistical analysis

Continuous data are presented as mean ± SD or 95% Confidence Interval (95% CI) or median and interquartile. Categorical variables are shown as percentage. To account for the non-independence among variables from each pair, to compare symptoms of anxiety, depression and PTSD among patients and family members, the McNemar test was used for categorical variables. For continuous variables, paired Student’s t-test and Wilcoxon signed-rank test were used. Correlation among hours spent at the ICU and HADS and IES scores was assessed by the Spearman’s rank correlation coefficient.

To evaluate repeated measures of HADS score, we adjusted a Generalized Linear Mixed Model, adding a random factor to account for the non-independence among the repeated observations for each individual. In this model, we also added another random intercepts to account for the correlation among each pair. Post-hoc analysis was used to compare three different groups (Patients, family members of patients who survived and family members of patients who deceased). To assess the correlation between HADS score within each pair we ran a Linear Mixed Model, considering random effects for each pair.

To identify factors associated with an increased IES score at 30-day, we first adjusted a univariate linear regression. After univariate analysis, variables with p values < 0.250 were selected to a backward stepwise selection procedure. The p values used as entry and removal criterion in the backward elimination were 0.05 and 0.10, respectively. To adjust the final model, we ran a Generalized Linear Mixed Model, adding random intercepts to account for the correlation among each pair.

A two-sided p-value ≤ 0.05 was considered statistically significant. The statistical analyses were performed using SPSS 19.0 software (Chicago, Il, US) and using R project software (www.r-project.org) version 3.0.2.

## Results

**Figure 1:**
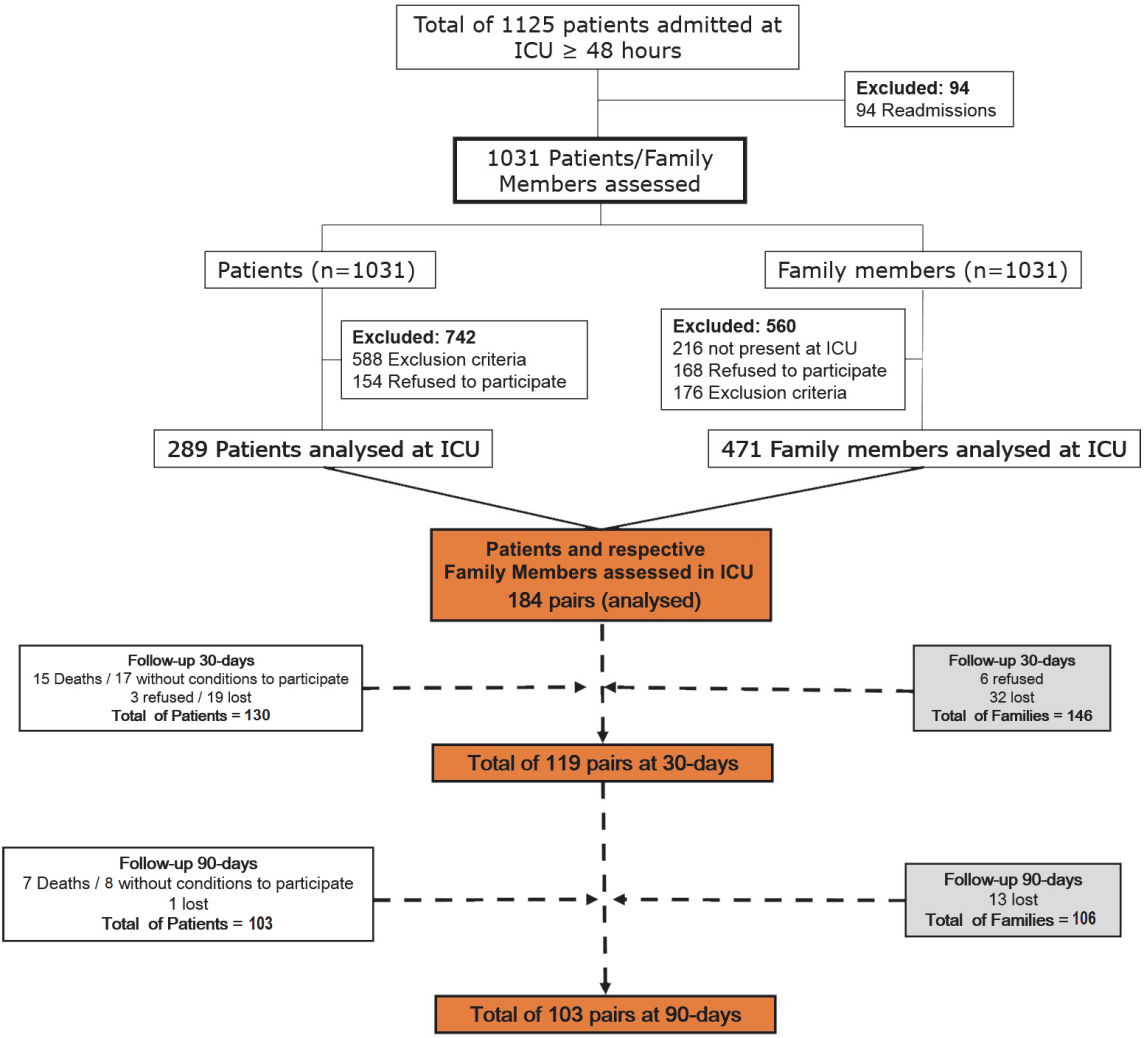
Flow chart of the study.

From March 2011 to March 2013, 1125 patients were admitted at the ICU ≥ 48 hours. Of this total, 471 family members and 289 patients were interviewed, resulting in 184 pairs on which the analysis was carried out. Reasons for non-inclusion are outlined in Fig. 1. Patients and family members were interviewed in a median of 3 (2–4) days after ICU admission.

For the entire cohort, the mean age was 60 ± 16 and 53 ± 13 years for patients (n = 289) and family members (n = 471), respectively. Patients had a mean SAPS 3 score of 46 ± 15 and a median of 4 [3–5] days of ICU stay. We observed that ICU mortality was 11.1%; at 30-day was 21.9% and at 90-day cumulative mortality was 28.6%. Regarding the 184 pairs analyzed, demographic characteristics were described in Table 1.

**Table 1:** Characteristics of patients and their respectively family members.

**Variables**	**Values**
**Patients (n = 184)**	
Age (Years)	59.3 ± 15.5 (18–92)
Gender female	67 (36.4)
College education	150 (81.5)
ICU length of stay (Days)	5.5 ± 5.5 (2–47)
SAPS 3	47.6 ± 15.7 (16–93)
Predicted mortality (%)	21 ± 20 (1–87)
SOFA	2.38 ± 2.7 (0–15)
Mechanical Ventilation >24 hours	46 (25)
Vasopressors	62 (33.7)
Renal Replacement therapy in ICU	22 (12)
Cancer	101 (54.9)
Delirium	11 (6.0)
ICU mortality	4 (2.0)
90-days mortality	22 (12.0)
**Family Members (n = 184)**	
Age (Years)	51.8 ± 13.4 (21–81)
Gender Female	146 (79.3)
Spouse	118 (64.1)
Offspring	46 (25.0)
Religious background	127 (69.0)
College education	137 (74.5)
Family staying time in the ICU (Hours/Day)	15.09 ± 6.89 (1–24)
Previous family experience with ICU	136 (73.9)

Values are presented as N (%) for categorical variables and mean ± SD (Range) for continuous variables.

Anxiety, depression and PTSD symptoms were described in Table 2 by the HADS and IES scores. HADS and its subscales scores significantly decreased over time for patients and family members. Patients scored lower values of anxiety and depression symptoms at 30 and 90-days, with the anxiety subscale as main determinant of this difference. Regarding IES score, at 30-days, patients and family members scored similar values, however family members had significantly higher values at 90-days (Table 2).

**Table 2:** Anxiety, depression and post-traumatic stress disorder symptoms among patients and family members.

	**HADS Total Score**	**HADS Subscale Anxiety score**	**HADS Subscale Depression score**	**IES score**
**Patients, mean (95% CI)**				
At ICU (n = 184)	10.7 (9.6–11.8)	7.0 (6.4–7.6)^a^	3.7 (3.1–4.3)	-
30-day (n = 119)	3.8 (3.0–4.6)^b^	1.9 (1.5–2.3)^c^	1.9 (1.4–2.4)^d^	6.5 (4.1–8.9)
90-day (n = 103)	2.0 (1.3–2.7)^e^	1.1 (0.7–1.4)^f^	0.9 (0.5–1.3)	1.5 (0.6–2.4)^g^
**Family Members, mean (95% CI)**				
At ICU (n = 184)	11.0 (9.9–12.2)	7.8 (7.1–8.5)^a^	3.3 (2.7–3.8)	-
30-day (n = 119)	7.3 (6.0–8.6)^b^	4.2 (3.5–4.9)^c^	3.2 (2.5–3.8)^d^	6.5 (4.7–8.3)
90-day (n = 103)	5.5 (4.1–6.9)^e^	3.1 (2.4–3.9)^f^	2.4 (1.7–3.1)	5.2 (3.2–7.3)^g^

Comparison between patients and Family members at each time point.

^a^ p = 0.016

^b^ p = 0.001

^c^ p<0.001

^d^ p = 0.076

^e^ p = 0.013

^f^ p = 0.002

^g^ p = 0.019

Comparison following time for patients: HADS total score and subscales: p<0.001; IES score p<0.001

Comparison following time for family members: HADS total score and anxiety subscale: p<0.001; HADS score depression subscale: p<0.001; IES score p = 0.052

HADS denotes Hospital Anxiety and Depression Scale and IES denotes Impact of Event Scale.

Through categorization of HADS score (> 10 points), patients and family members had comparable symptoms of anxiety (26.1% vs. 33.2%, respectively; p = 0.117), depression (12% vs. 22.2%, respectively, p = 0.597) or both (8.7% vs. 9.8%, respectively, p = 0.99) during ICU stay. However, at 30-days fewer patients presented with anxiety symptoms than family members (0.8% vs. 7.6%, respectively; p = 0.008) and comparable depression symptoms (3.4% vs. 6.7%, respectively; p = 0.344). Both symptoms were found only in family members at 30-days (4.2%). At 90-days, only family members had anxiety symptoms (1.9%) and depression symptoms were similar among patients and family members (1.0% vs. 3.9%, respectively; p = 0.375). Regarding PTSD symptoms, after categorization of the IES score, patients and family members presented similar suffering at 30-days (5.9% vs. 2.5%, respectively; p = 0.344). At 90-days, only family members of patients who deceased had PTSD symptoms (4.9%).

**Figure 2:**
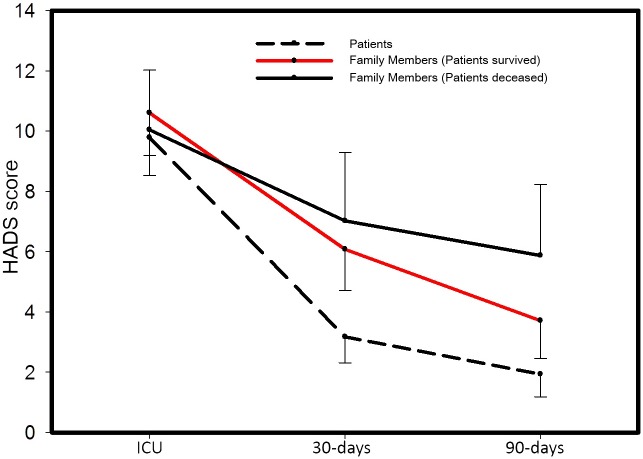
HADS score over time distributed between groups.

**Figure 3:**
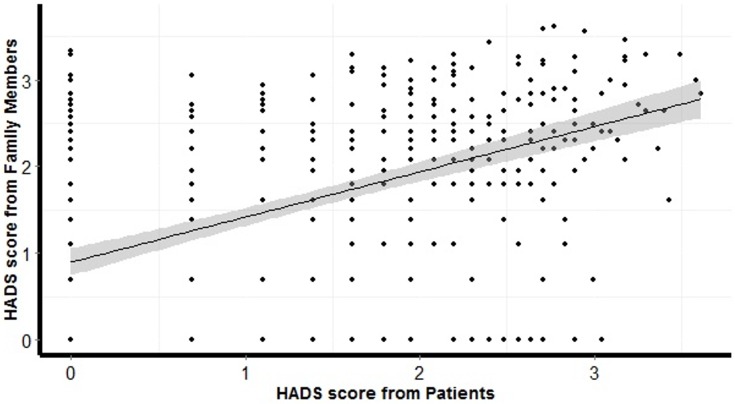
Correlation between patients and family members anxiety and depression scores considering values from ICU, 30 and 90-days after ICU discharge. HADS denotes Hospital Anxiety and Depression Scale. The x- and y-axis were logarithmic transformed.

Considering HADS score over time with repeated assessments, we found that anxiety and depression symptoms varied significantly between groups over time (p<0.001), Fig. 2. Post-hoc analysis showed that patients presented with less symptoms of anxiety and depression at 30 and 90-days than family members of patients who survived and who deceased (p<0.001). At 90-days, family members of patients who deceased presented higher anxiety and depression symptoms when compared with family members of patients who survived (p = 0.048). As previously hypothesized, symptoms of anxiety and depression were correlated between patients and family members in a positive direction (r = 0.5186, p = 0.001, Fig. 3).

There was no correlation between family members hours visiting and HADS score in the ICU (rho = 0.111; p = 0.134), at 30 (rho = −0.005; p = 0.956) and 90-days (rho = −0.004, p = 0.964). Moreover, there was no correlation between hours visiting at ICU and IES score at 30-days (rho = 0.023; p = 0.784) and 90-days (0.012; p = 0.888) for family members.

A multivariate model was constructed for risk factors associated with PTSD symptoms. Higher risk of PTSD was associated to symptoms of anxiety and depression during ICU stay, also with younger and female patients. Renal replacement therapy was the highest risk factor for patients who showed PTSD symptoms after ICU discharge. Interestingly, female gender and renal replacement therapy had a positive interaction in this model, Table 3. In the case of family members, the highest risk factors to PTSD symptoms were the younger patients and those who died in the ICU. Regarding family characteristics, symptoms of anxiety and depression during ICU stay were predictor of PTSD, while religiosity was a protective factor, Table 3.

**Table 3:** Multivariate analysis for factors associated with the IES score for patients and family members at 30 days.

**Model for Patients**			
**Variables**	**Beta (SE)**	**IC 95%**	**P value**
**Patient**			
Age (years)	− 0.257 (0.06)	− 0.368; − 0.146	<0.001
Female*	4.909 (1.97)	1.052; 8.767	<0.001
Renal Replacement therapy in ICU*	15.343 (3.10)	9.272; 21.413	<0.001
Symptoms of anxiety and depression at ICU	10.936 (3.54)	3.999; 17.872	0.002
**Patient-Family related**			
Married	4.467 (2.58)	− 0.596; 9.530	0.084

**Model for Family Members**			
**Variables**	**Beta (SE)**	**IC 95%**	**P value**
**Patient related**			
Age (years)	− 0.123 (0.05)	− 0.232; − 0.014	0.027
Death at ICU	13.559 (7.24)	− 0.637; 27.756	0.061
**Family**			
Symptoms of anxiety and depression in ICU	13.574 (3.07)	7.567; 19.581	<0.001
Religious belief	− 3.900 (1.79)	− 7.411; − 0.389	0.029

* This model taken account an interaction term between Female gender and Renal Replacement Therapy.

## Discussion

This study intended to compare symptoms of anxiety, depression and post-traumatic stress of patients and their respective family members. The central point of this study is that we assessed patients with their families, in exact patient-family pairs at three time points (during ICU stay, at 30 and at 90-days after discharge). To our knowledge, few studies evaluated pairs at multiple points and in most, the initial evaluations occurred within two months of ICU discharge, and at varying intervals of up to eight years [1,2].

Our main finding was that family members of ICU patients suffer more anxiety, depression and post-traumatic stress symptoms than the patients. Furthermore, family members of patients who deceased scored higher for these symptoms. According to current literature, family members suffers more during and post-ICU, mainly when the loved one becomes critically ill or die [4–8,18–23].

Our study supports the hypothesis that patients and family members that initially had symptoms of anxiety and depression were more vulnerable to post-traumatic stress symptoms [2,7], thus raising a possibility to identify and provide a better and earlier support. To having a loved one die is a strongly stressful event and we found that it was a predictor of PTSD symptoms. As such, we detected that there is a correlation of anxiety and depression symptoms between patients and family members in a positive direction. It was considered relevant that the anxiety, depression and post-traumatic stress symptoms in patients lessened significantly after three months, whereas in family members they persisted three months after ICU discharge. Patient recovery varies worldwide. We agree with the hypotheses that family members retain more memories of the ICU experience than the patients, who often have a vague remembrance of their time in the ICU [22]. Many studies did not find a difference in the IES score in patients over time, but some found that anxiety and depression scores were significantly reduced over time [2,27].From the 184 pairs, follow-up of 20 patients and 45 family members was lost, which may be a matter of concern. Subjects who refused to participate or lost the follow-up could be suffering and therefore avoided contact with the memories and might have been under risk of PTSD.

Regarding patients, the stronger predictors of PTSD were RRT and symptoms of anxiety and depression in the ICU. We can speculate that RRT was a marker of severity, however the psychosocial aspects of patients under dialytic therapy is an important aspect, predominantly centered on depressive disorders, which is associated with increased risk of mortality and poor health-related quality of life [28,29].

Previous studies alert to the need of interventions to alleviate emotional distress in patients and their family members [7,8,20,22]. Indeed, a number of studies have indicated that relatives who receive better information and psychological assistance during their permanence in an ICU, mainly those of dying patients, presented less anxiety, depression and post-traumatic symptoms [8,30]. We strongly advise an early psychological support during ICU, principally when loved ones are at risk of death [30]. Our study holds new data about the correlation between symptoms of patients and their respectively family member. This finding should be considered when both are facing psychological distress. We believe that diagnosis of anxiety and depression symptoms in patients should bring about close attention to the family member as well as the opposite.

Cultural aspects are a determinant factor of the psychological ones of patients and family members. We found that older and male patients, together with religious beliefs were protective factors regarding development of post-traumatic stress. In previous studies, patients with a high level of spirituality were less likely to be anxious or depressed [31,32]. Religious beliefs usually influence the existential well-being, anxiety and depression. Moreover, Brazil is a country of strong Catholic tradition and, in keeping with findings from other studies, people with religious beliefs show a greater acceptance of death and suffer less anxiety and depression [33].

Our study was carried out in an open visit ICU, which allows access to family members at all times (24 hours/day). The open visit ICU is a scenario poorly studied in current literature [12,13,34]. In this setting, our results were similar to current literature about patient and family member comparisons regarding anxiety, depression and PTSD symptoms. Young found that ICU patients’ relatives reported significantly more symptoms of anxiety than did ICU patients at three months [22]. In a sample of patients with chronic obstructive pulmonary disease, Miranda et al. evaluating these symptoms in both patients and family members, found similar results showing that prevalence of anxiety and depression symptoms were high at ICU discharge and decrease over time. The authors also reported higher rates of anxiety, depression and PTSD symptoms in relatives than in patients [23].

We believe that an open visit ICU may bring many benefits to family members and patients to diminish their symptoms albeit this help alone is not sufficient. We know that family conference can improve communication and is important to acknowledge the emotions that come up in these discussions [8,10]. We defend the need for a family conference and the presence of a psychologist for early identification of those at risk of suffering with these symptoms to better support them. We did not find any correlation between hours spent by family members in the ICU and HADS or IES scores. However, it is also possible that our sample was not sufficient to correctly address this issue. Furthermore, as the quantification of hours visiting at the ICU was reported by the family member and not audited, it could be biased.

The main limitation of this study is its single center design, performed in a private Brazilian ICU. This model and organization of an open visit ICU with private rooms is not representative of all Brazilian ICUs, although many are nowadays changing to facilitate family visiting [12–16]. In Brazil, the majority of ICUs is in public health institutions, with restrictive visiting hour’s policy. Another limitation includes the high percentage of non-included patients, because of their poor condition at ICU admission. Although we have a large number of pairs (patient and family member) enrolled in the study, this loss can underestimate the frequency of symptoms of anxiety, depression and PTSD. We did not evaluate the revised version of IES score (IES-R), which has additional items related to the hyperarousal symptoms, because it had not yet been cross-validated for the Brazilian Portuguese when we began data collection. Additionally, HADS and IES are not tools for diagnosis but instead for detection of symptoms indicating a risk of anxiety, depression and of PTSD, respectively. The lack of a Structured Clinical Interview does not allow us to confirm the incidence of diagnosed anxiety, depression and PTSD. Finally, dichotomizing continuous data is accompanied by important power loss of the data in comparison with their continuous values [35].

## Conclusions

Family members of ICU patients suffered more than patients, especially when the loved one died. Symptoms of anxiety, depression and post-traumatic stress persisted in family members over time. Contrariwise, in patients these symptoms decreased in three months. We recommend more support to patients and family members who presented anxiety and depression during ICU stay because of the higher risk of suffering from post-traumatic stress symptoms. In addition, we recommend that this support may be extended to the follow-up. Finally, our study was conducted in an open visit ICU that allows access to the family at all times (24 hours). This potential benefit for patients and family members and the impact on emotional disorders is an important domain for future research.
